# Supplementary material for: Impact of maternal iron and zinc intake on low birth weight risk: a nested case—control study
Source: PeerJ. 2025 Aug 18;13:e19896. doi: 10.7717/peerj.19896 (PMC12369602; doi:10.7717/peerj.19896)
Supplement: Supplemental Information 1 [file peerj-13-19896-s001.pdf]

STROBE Statement—checklist of items that should be included in reports of observational studies

	Item No	Recommendation
**Title and abstract**	1	(*a*)Located on lines 1 through 2 of the manuscript
		(*b*) Located on lines 26 through 29 of the manuscript
**Introduction**		
Background/rationale	2	Located on lines 33 through 61 of the manuscript
Objectives	3	Located on lines 61 through 63 of the manuscript
**Methods**		
Study design	4	Present key elements of study design early in the paper
Setting	5	Located on lines 66 through 67 of the manuscript
Participants	6	(*a*)Located on lines 66 through 74 of the manuscript
		(*b*) Located on lines 72 through 74 of the manuscript
Variables	7	Located on lines 77 through 101 of the manuscript
Data sources/ measurement	8*	Located on lines 103 through 109 of the manuscript
Bias	9	Located on lines 111 through 118 of the manuscript
Study size	10	Located on lines 66 through 67 of the manuscript
Quantitative variables	11	Located on lines 120 through 144 of the manuscript
Statistical methods	12	(*a*)Located on lines 120 through 144 of the manuscript
		(*b*)Located on lines 136 through 144 of the manuscript
		(c)Located on lines 70 through 73 of the manuscript
		(d)Located on lines 95 through 101 of the manuscript
		(e)Located on lines 134 through 136 of the manuscript

Continued on next page

**Results**		
Participants	13*	(a) Located on lines 68 through 74 of the manuscript
		(b) Located on lines 70 through74 of the manuscript
		(c)
Descriptive data	14*	(a)Located on lines 111 through 118 of the manuscript
		(b) Located on lines 68 through 74 of the manuscript
		(c) *Cohort study*—Summarise follow-up time (eg, average and total amount)
Outcome data	15*	*Cohort study*—Report numbers of outcome events or summary measures over time
		Located on lines 147 through 188 of the manuscript
		Cross-sectional study—Report numbers of outcome events or summary measures
Main results	16	(*a*) Located on lines 147 through 188 of the manuscript
		(*b*) Located on lines 89 through 90 and 103 through 109 of the manuscript
		(*c*) If relevant, consider translating estimates of relative risk into absolute risk for a meaningful time period
Other analyses	17	Located on lines 156 through 202 and 133 through 135 of the manuscript
**Discussion**		
Key results	18	Located on lines 156 through 202 of the manuscript
Limitations	19	Located on lines 264 through 266 of the manuscript
Interpretation	20	Located on lines 329 through 334 of the manuscript
Generalisability	21	Located on lines 229 through 332 of the manuscript
**Other information**		
Funding	22	This work was supported in part by Gansu Provincial Science and Technology Department Grant (No.22JR5RA633) and the Science and Technology Department Grant of Lanzhou City (No.2022-5-85) .

*Give information separately for cases and controls in case-control studies and, if applicable, for exposed and unexposed groups in cohort and cross-sectional studies.

Note: An Explanation and Elaboration article discusses each checklist item and gives methodological background and published examples of transparent reporting. The STROBE checklist is best used in conjunction with this article (freely available on the Web sites of PLoS Medicine at http://www.plosmedicine.org/, Annals of Internal Medicine at http://www.annals.org/, and Epidemiology at http://www.epidem.com/). Information on the STROBE Initiative is available at www.strobe-statement.org.
